# Notes and News

**Published:** 1905-03-25

**Authors:** 


### Notes and News.

The Queen has consented to be patron of the University College Hospital Ladies' Association.

Princess Christian has become the president of the Royal Maternity Charity of London, a post which has been vacant since the death of the late Duke of Argyll.

Princess Louise Augusta of Schleswig-Holstein will open the wards for whooping-cough at the East London Hospital for Children on Saturday, April 8th.

At Glastonbury a man was summoned for exposing his son when suffering from scarlet fever. The boy was employed to deliver turf, although the medical officer of health and the sanitary inspector had warned the father not to allow him to mix with others until pronounced free of infection. The father—as the responsible person—pleaded ignorance of the disease and of the law, and the chairman of the county sessions, in convicting him, pointed out that he was liable to a penalty of £5, but "as you are apparently not troubled with a surplus of brains in your head," he said, "we will let you off with a fine of 10s. and costs."

In response to petitions presented by the London School of Tropical Medicine in February 1904, the Senate of the University of London has decided to institute a sixth branch of study—viz. tropical medicine, in which candidates may be examined for the M.D. degree, and has admitted the London School of Tropical Medicine as a school of the University in the subject of tropical medicine. The University of the metropolis of the Empire has thus recognised its Imperial character and has brought itself into line with Cambridge and other Universities in regard to the importance of the health of Europeans and natives in the great tropical dependencies of the nation. These facts will be emphasised at the banquet over which Mr. Chamberlain will preside on behalf of the London School of Tropical Medicine on May 10th.

The Italian Ambassador visited the Italian Hospital on Saturday to present to the hospital, on behalf of the committee of management, a portrait of Mrs. Angiola Ortelli, honorary lady superintendent, and widow of the founder of the institution. The occasion commemorated the 21st year of the hospital's existence.

An infectious diseases hospital for Horwich, Westhoughton and Blackrod has been opened at Horwich. It consists of three ward blocks for 46 patients, administration block, laundry, and disinfection block. The ward blocks are for typhoid, scarlet fever, and diphtheria respectively. The cost has been about £471 per bed.

The late Mrs. Jane Begley, of Hammersmith, has bequeathed £1,000 to augment the legacy from her late husband, Dr. Chapman Begley, to Trinity College, Dublin, for the endowment of four medical scholarships. She also bequeathed £500 to the Royal College of Physicians and to All Saints' Convalescent Home, Eastbourne, and the West London Hospital, and smaller sums to the Royal Hospital for Incurables and St. Mary's Hospital. The late Mr. Edward Crossley, of Halifax, bequeathed £1,000 to the Halifax Royal Infirmary and £500 to the Royal Isle of Wight Hospital.

It is intended to establish a small-pox hospital for the district of Nuneaton and Bulkington. The site proposed is near Burton Hastings, and it was mentioned at the meeting called to consider the proposals that numbers of school children must pass within 50 yards of the hospital, which appeared to be held as a safe distance by most present. Considering that the problem of the aerial dissemination of small-pox has not been by any means disposed of, we cannot think that 50 yards is a long enough distance between a small-pox hospital and the high road. The extent to which medical men differ on the point was illustrated at a recent conference in connection with the Royal Sanitary Institute held at Newcastle-on-Tyne, but until definite data are obtainable, practice had better err on the side of excessive caution.

Mr. Edgar Speyer has offered a prize of £100 and a silver cup for the best essay on "The Economical Management of an Efficient Voluntary Hospital." The Prince of Wales has consented to present the prize, and the competition is limited to the paid secretaries and assistant secretaries of voluntary hospitals in the United Kingdom and Ireland. We feel it to be a mistake to limit the competition to hospital secretaries, for many of the economies enforced throughout the hospital system owe their origin to the matrons or medical superintendents, amongst whom some of the most economical and knowledgeable of hospital economists are to be found. Mr. Speyer's liberality should be made to yield as fruitful a harvest as possible by the competition being thrown open to all hospital officials. In this way every system which enforces economy could be made to yield its quota of help for the general good of all the hospitals. Particulars and conditions may be obtained from the hon. secretaries of King Edward's Hospital Fund, 81 Cheapside, E.C.

